# A Case-Based Overview of the Role of Radiological Imaging in Emergency General Surgery

**DOI:** 10.7759/cureus.21986

**Published:** 2022-02-07

**Authors:** Jane Kilkenny, TSW Greensmith, Waseem Hameed, Simon Gill, Sarah Hassan

**Affiliations:** 1 General Surgery, Royal Victoria Hospital, Belfast, GBR; 2 Orthopaedic Surgery, Darlington Memorial Hospital, Darlington, GBR; 3 Colorectal Surgery, Wexham Park Hospital, Slough, GBR; 4 Radiology, Wexham Park Hospital, Slough, GBR; 5 Colorectal Surgery, John Radcliffe Hospital, Oxford, GBR

**Keywords:** medical education, surgical imaging, surgical radiology, surgical education, emergency general surgery

## Abstract

This article aims to give an overview of some of the common conditions seen in emergency general surgery and the recommended choice of imaging. For junior doctors, choosing the correct imaging modality can be difficult so we aim to provide a summary of the evidence behind radiology for emergency general surgery. Four of the most important acute surgical conditions were chosen, alongside abdominal aortic aneurysm. A literature search was carried out to review the most up-to-date evidence regarding imaging choices. Cases were chosen from everyday practice to put the imaging into context. This article gives an overview of the most common imaging modalities used in emergency general surgery. It can be used by medical students and junior doctors to help understand the reasoning behind imaging choices on the acute surgical take.

## Introduction and background

Abdominal imaging is a critical step in the investigation and management of patients presenting with abdominal pain. Plain radiographs, ultrasound (USS), computed tomography (CT) and magnetic resonance imaging (MRI) are among the many imaging modalities used to investigate abdominal symptoms to allow swift and appropriate diagnosis and management. For junior doctors, there is often uncertainty when choosing the initial imaging modality and this can cause unnecessary delays while awaiting senior input. It is important for all grades of doctors to understand the basic imaging indications for common surgical presentations to ensure prompt treatment can commence. This article gives an overview of common conditions seen in emergency general surgery and provides recommendations for the most appropriate imaging pathways for patients presenting with abdominal pain. Four very common acute general surgical conditions were chosen, alongside abdominal aortic aneurysm, which although does not occur as regularly, but is extremely important to be aware of. A literature review was carried out to explore the most up-to-date evidence regarding imaging choices and cases were chosen from everyday practice to put the imaging into context.

## Review

Case one

A 28-year-old female attends the emergency department with a two-day history of umbilical pain radiating to the right iliac fossa. The pain has a gradual onset and is constant. There is associated nausea, anorexia and low-grade fever. Although the sensitivity and specificity of diagnosing appendicitis on USS are lower than that of CT, the absence of ionising radiation lends USS to be the investigation of choice, especially in paediatrics and young adults, where there is diagnostic uncertainty [1]. A systematic review in 2007 found the sensitivity and specificity of USS in diagnosing appendicitis to be 83.7% and 95.9%, respectively, compared to CT which was 93.4% and 93.3%, respectively [2]. USS can identify many signs of acute appendicitis including a diameter of the appendix, increased wall thickening of the appendix, an appendicolith, surrounding free fluid, an abscess, or an appendicular mass [2].

**Figure 1:**
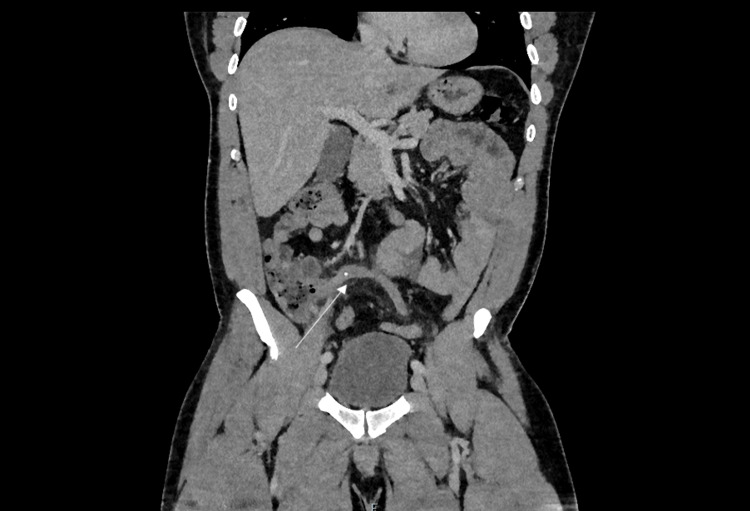
CT abdomen/pelvis showing a dilated, fluid-filled appendix with an appendicolith at the proximal aspect in keeping with acute appendicitis.

The differential diagnosis of right iliac fossa pain is wider in the population above the age of 40 so CT is often used first-line in these patients to rule out other causes including right-sided diverticulitis or malignancy [2]. A typical CT representation of appendicitis is shown in Figure 1.

Case two

A 68-year-old male attends the emergency department with severe left iliac fossa pain. He has known diverticulosis that was seen on colonoscopy two years ago. The pain is constant, gradually worsening and radiating across his lower abdomen. On examination, he has peritonism in his left iliac fossa. He has raised inflammatory markers.

Diverticular disease is increasingly common across the Western world. Studies show a prevalence of colonic diverticula in up to 50% of people over 50 years of age with 10-25% developing complications such as inflammation or diverticular bleeding [3]. The management of acute diverticulitis depends on the severity and can be classified as complicated or uncomplicated.

CT is the imaging of choice for diverticulitis in the acute, symptomatic stage. It is used to establish the diagnosis, its severity and any associated complications. It classifies the severity of complicated diverticulitis radiologically using various methods. The most used method is the Hinchey Classification score, as seen in Table 1. In stages one and two, there is a contained abscess either localised (Hinchey 1) or in the pelvis (Hinchey 2). In the most severe cases, abscess perforation leads to purulent peritonitis (Hinchey 3) and diverticula rupture to faecal peritonitis (Hinchey 4) [4].

**Table 1:** Hinchey classification of acute complicated diverticulitis.

Hinchey Stage	Complication
1	Localised abscess (para-/mesocolic)
2	Pelvic abscess
3	Purulent peritonitis
4	Faeculant peritonitis

**Figure 2:**
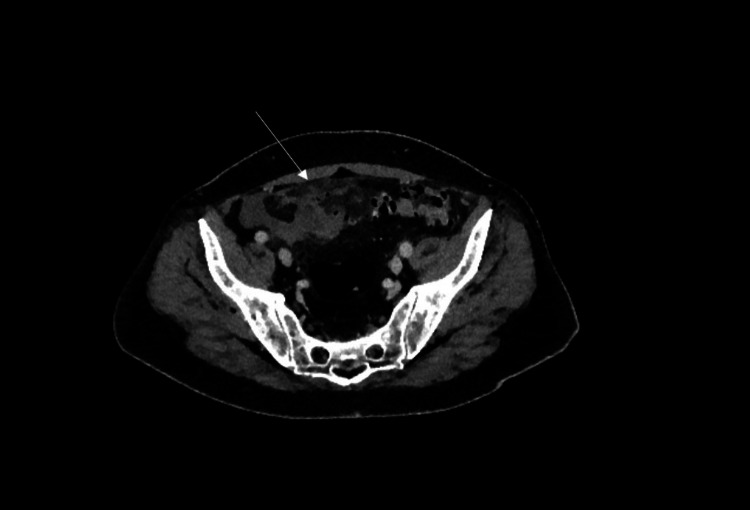
CT abdomen/pelvis showing significant inflammatory stranding surrounding diverticula of the sigmoid colon with pockets of free extra-luminal air.

Common findings on CT include pericolic stranding, segmental thickening of the bowel wall, abscess formations or diverticular perforation which will show extravasation of gas and fluid into the peritoneal cavity. An example is shown in Figure 2. After the first episode of acute diverticulitis, a follow-up colonoscopy should be performed after six weeks to directly visualise the diverticular disease and to exclude malignancy.

Case three

A 48-year-old female presents with a three-day history of constant right upper quadrant (RUQ) pain radiating to her shoulder tip. She feels generally unwell and feverish. Her inflammatory markers are raised.

The first-line imaging for RUQ pain is ultrasound imaging [5]. It is the preferred imaging modality due to its high sensitivity for detecting gallstones (87% as opposed to 60% for CT, and 97% for MRI), low cost, easy accessibility and lack of ionising radiation [6]. USS can diagnose cholecystitis by illustrating localized gallbladder tenderness, gallbladder wall thickening, gallbladder stones and pericholecystic fluid [5]. USS may also show common bile duct (CBD) dilatation and occasionally CBD stones.

**Figure 3:**
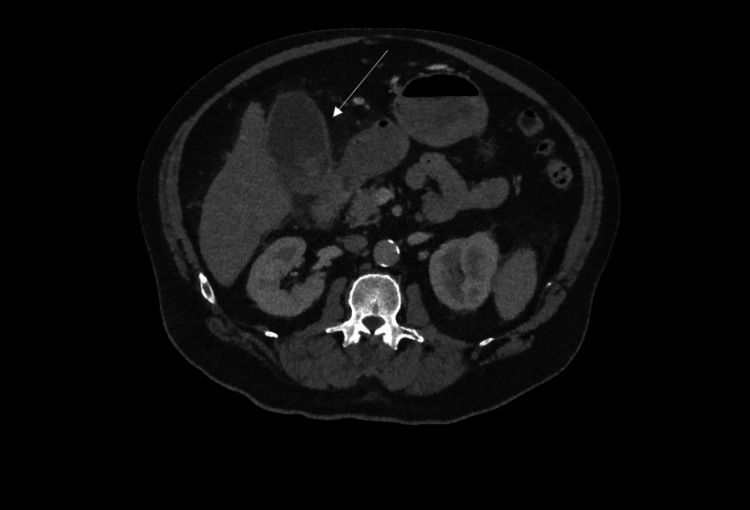
CT abdomen showing a thick-walled, distended gallbladder, in keeping with acute cholecystitis.

Once cholecystitis is confirmed on USS and the patient is responding to initial management, no further imaging is required. However, USS has limited value in the evaluation of complications from acute cholecystitis so further imaging may be required and CT or MRI are the modalities of choice [7]. Findings on CT can include a thick-walled gallbladder, surrounding inflammatory stranding, gallbladder perforations and collections or abscesses. Figure 3 shows a typical view of acute cholecystitis on CT imaging.

Further imaging is usually required if USS shows a dilated CBD. Magnetic resonance cholangiopancreatography (MRCP) is an imaging technique used to visualise the intra- and extrahepatic biliary tree and pancreatic ductal system. It can be used to determine the cause of the duct dilatation, as it can visualise stones and strictures [8].

If a stone is impacted in the CBD, progression to endoscopic retrograde cholangiopancreatography (ERCP) is required before cholecystectomy. This allows direct visualisation and definitive management of the stone. Any patient who has had complications from gallstones and is fit for surgery should be offered a cholecystectomy. The timeline varies depending on the condition. Laparoscopic cholecystectomy should be performed on the index admission for gallstone pancreatitis however for acute cholecystitis, it should be offered either within the first 72 hours of pain or after six weeks as an urgent outpatient.

Case four

A 34-year-old female presents to the emergency department with epigastric pain radiating through to her back. She is vomiting and initial investigations show high inflammatory markers and raised amylase. She has a tender epigastrium on examination and prefers to be sat up to reduce the pain. She has recently been discharged from the hospital with an episode of acute cholecystitis. Her clinical condition continues to deteriorate despite normal supportive therapy over the following few days.

Alcohol and gallstones are the two most common causes of pancreatitis in the adult population [9]. Most patients with pancreatitis endure only a mild course of the disease with supportive therapy being the mainstay of treatment and recovery within one week is usually seen [10]. A small proportion of patients go on to have severe and life-threatening courses of pancreatitis.

The International Association of Pancreatology guidelines, set in 2013, advise monitoring the presence of systemic inflammatory response syndrome (SIRS) or organ failure on admission and for 48 hours to predict the development of a severe course of the disease [11,12]. There are predictive scoring systems, such as the Modified Glasgow Acute Pancreatitis Severity Score, APACHE II (Acute Physiology and Chronic Health Evaluation II) and Ranson’s Criteria for Pancreatitis Mortality, however, these have proven to be neither superior nor inferior to SIRS [13].

If typical clinical and laboratory findings are present, imaging is not required to confirm the diagnosis of acute pancreatitis [12]. Abdominal ultrasound is used to investigate the aetiology, by looking for gallstones. The International Association of Pancreatology outlines guidance for the indication for initial CT assessment of acute pancreatitis, which has been adopted by the Royal College of Radiologists as audit standards [11]. These standards are in the case of diagnostic uncertainty, for confirmation of severity based on clinical predictors of severe acute pancreatitis and failure to respond to conservative treatment or in the setting of clinical deterioration.

**Figure 4:**
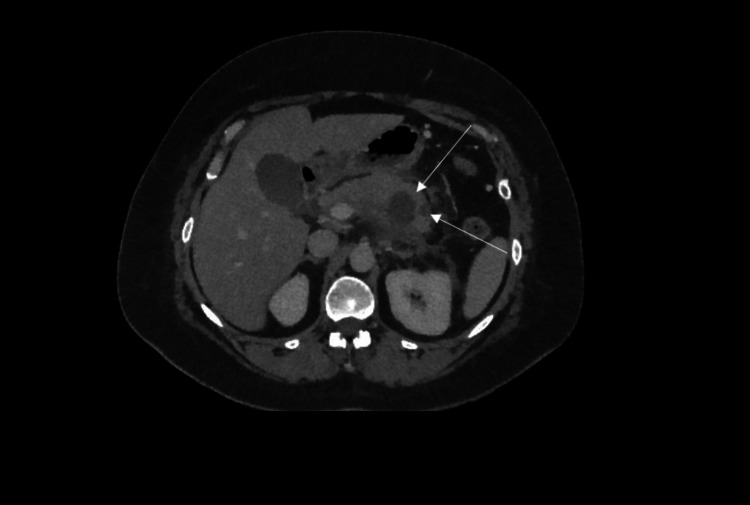
CT abdomen showing acute pancreatitis with peri-pancreatic inflammatory stranding and two pseudocysts in the tail of the pancreas.

**Figure 5:**
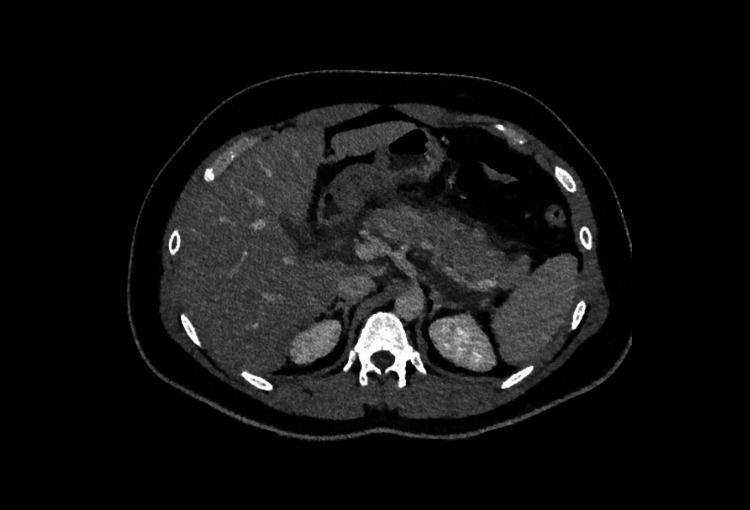
CT abdomen showing peri-pancreatic inflammatory stranding in keeping with acute severe pancreatitis, with no evidence of necrosis.

Initial CT assessment optimal timing is at least 72-96 hours after onset of symptoms. CT can comment on the presence of pancreatic necrosis as well as the site and presence of free fluid and peri-pancreatic fat stranding. Other local complications can be visualised by CT: fluid collections, pseudocyst formation, abscesses, haemorrhage, venous thrombosis and pseudoaneurysm. In the majority of patients with pancreatitis, routine CT imaging is not indicated as there is no evidence that early CT improves clinical outcomes [11]. Figures 4-5 show typical features of acute pancreatitis.

Patients with mild biliary pancreatitis should be offered a cholecystectomy on the index admission. However, in a case with severe biliary pancreatitis, patients with peri-pancreatic collections should have delayed cholecystectomy until the collections resolve or if they persist beyond six weeks.

Case five

A 62-year-old male presents with profuse vomiting, absolute constipation and abdominal pain. He has a grossly distended abdomen which is tender and tympanic on examination. His past medical history includes an open appendicectomy performed 40 years ago for perforated appendicitis. Initial investigations reveal a mild acute kidney injury.

Bowel obstruction is a common presentation to the acute surgical unit, with the most common cause of small bowel obstruction being intraperitoneal adhesions (70% of cases) [14]. This patient has a history of open abdominal surgery, therefore, adhesional small bowel obstruction is the most likely, but not the only possible aetiology. Important initial management includes insertion of a nasogastric tube, intravenous fluid resuscitation and accurate fluid balance monitoring.

Abdominal x-ray is commonly used as the initial radiological investigation for patients with acute abdominal pain, however, studies have shown that abdominal x-rays are diagnostic in only 50-60% of cases [15]. There is literature addressing the poor sensitivity of plain abdominal x-rays [16,17]. This has led to some recommending CT as the first-line imaging in acute abdominal pain [18]. An abdominal x-ray can still be beneficial in the diagnosis of bowel obstruction and signs that can be seen on plain x-ray imaging include small bowel loops dilated greater than 3 cm, thickened bowel wall, collapsed colon or fluid-air levels.

**Figure 6:**
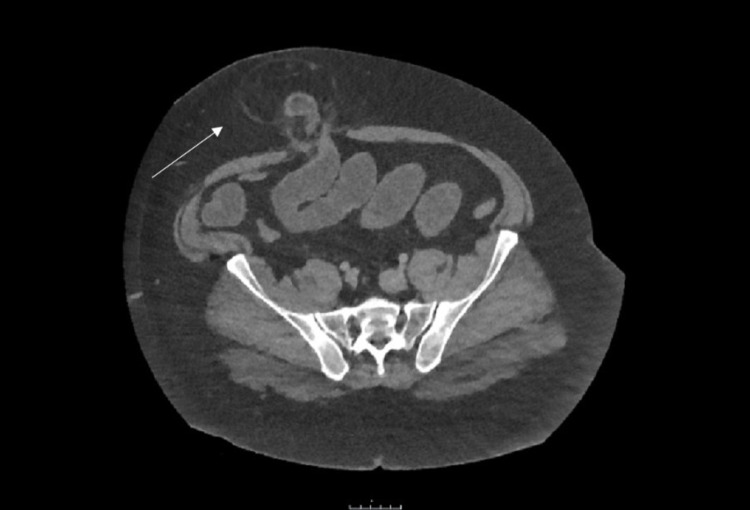
CT abdomen showing an axial view of paraumbilical hernia containing bowel, causing proximal small bowel dilatation.

**Figure 7:**
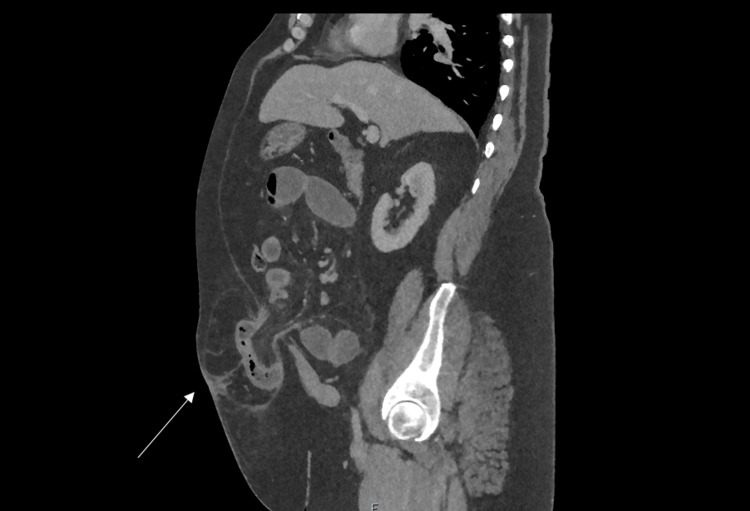
CT abdomen showing a sagittal view of a paraumbilical hernia containing bowel, causing small bowel obstruction.

Contrast-enhanced CT imaging is the preferred higher imaging. Along with confirming dilation of the small bowel, a transition point can often be identified; along with other causes of small bowel obstruction and can often identify if there is associated strangulation. Contrast-enhanced CT sensitivity for the identification of intestinal ischaemia is as high as 90% [15]. Causes of small bowel obstruction include adhesions, incarcerated abdominal wall and groin hernia, as seen in Figures 6-7, and internal hernia.

Small bowel obstruction secondary to an incarcerated abdominal wall hernia warrants urgent reduction, usually in the form of surgical repair. Adhesional small bowel obstruction can usually be settled with non-operative management (90% of cases) but a small proportion of patients will need to undergo definitive surgical intervention, ranging from band adhesiolysis to a small bowel resection [19]. Therefore, it is important to determine the cause, usually by CT, which is also useful for operative planning.

Case six

A 73-year-old male presents to the emergency department feeling unwell and complaining of severe back pain. His initial observations include a blood pressure of 90/60 mmHg and HR 89. He has a past medical history of hypertension, peripheral vascular disease, atrial fibrillation and he smokes 40 cigarettes a day. He takes bisoprolol regularly. On examination, he has a slightly tender abdomen, and a central pulsatile mass is palpated.

A ruptured abdominal aortic aneurysm has a mortality rate of over 90% [20]. It is one of the most fatal surgical emergencies. Only 25-50% of patients present with the classic triad of back pain, hypotension and a pulsatile mass [20]. A classic cause of misdiagnosis is a patient presenting with symptoms mimicking a renal stone.

**Figure 8:**
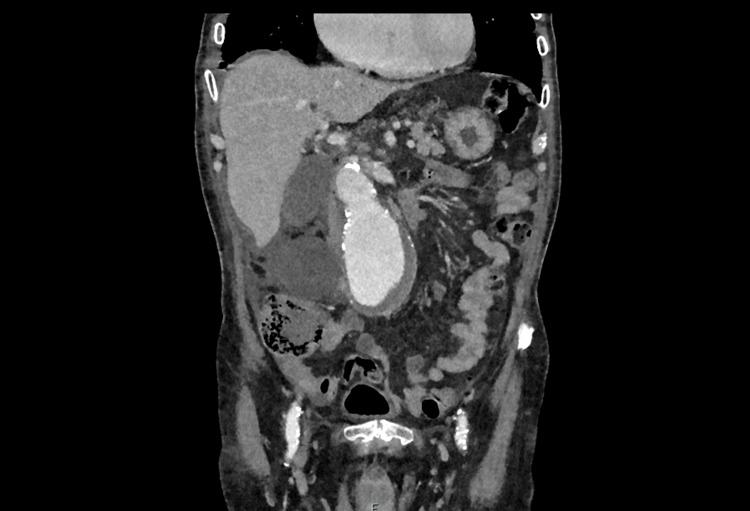
CT angiogram showing a coronal view of a large 7.5 cm infrarenal abdominal aortic aneurysm with incidental right-sided large renal cyst.

**Figure 9:**
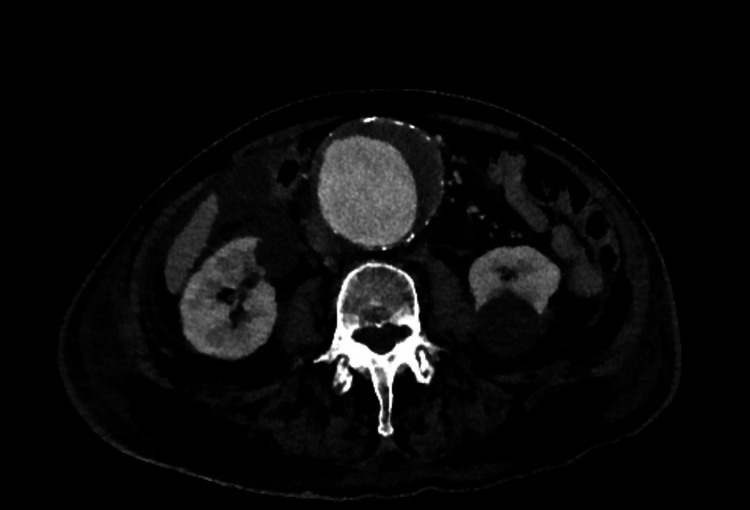
CT angiogram showing an axial view of a large 7.5 cm infrarenal abdominal aortic aneurysm with incidental bilateral renal cysts.

Emergency contrast-enhanced triple-phase CT scanning is indicated for the investigation of a potentially ruptured abdominal aortic aneurysm (AAA) [21]. CT is also used for planning repair, whether endovascular or open repair. Figures 8-9 show an example of a large aneurysm, found incidentally.

There is currently a screening program running in the UK for AAA. In England, men aged 65 are offered screening using an abdominal ultrasound scan. The diameter of the aorta determines the next steps in the patient pathway (Table 2). This is based on the risk of AAA rupture and aneurysm size (Table 3) [22].

**Table 2:** UK Abdominal Aortic Aneurysm Screening Surveillance Programme

Aortic Diameter (anterior-posterior)	Re-scanning Interval
<3 cm	Discharged from programme
3-4.4 cm	Annual surveillance programme
4.5-5.4 cm	3-monthly surveillance programme
>5.5 cm	Referral to vascular surgeon

 

**Table 3:** Estimated annual rupture risk of abdominal aortic aneurysm AAA: abdominal aortic aneurysm

AAA diameter (cm)	Rupture risk (%/year)
<4	0
4-5	0.5-5
5-6	3-15
6-7	10-20
7-8	20-40
>8	30-50

## Conclusions

This article provides an overview of common conditions that present on the acute surgical take. Understanding the indications for imaging and the rationale behind the choice of imaging is fundamental for junior doctors and medical students. This knowledge is vital for both day-to-day practice and examinations, and the evidence presented in this article can be utilised to further and improve your clinical practice.
